# Long‐Term Sperm Storage in a Superfetatious Live‐Bearing Fish (*Poeciliopsis gracilis*, Poeciliidae)

**DOI:** 10.1002/ece3.72086

**Published:** 2025-09-04

**Authors:** T. R. Ernst, R. M. H. W. Hogers, P. J. C. Kwant, A. Korosi, J. L. van Leeuwen, A. Kotrschal, B. J. A. Pollux

**Affiliations:** ^1^ Department of Animal Sciences, Experimental Zoology Group Wageningen University Wageningen the Netherlands; ^2^ Swammerdam Institute of Life Sciences, Center for Neuroscience, Brain Plasticity Group University of Amsterdam Amsterdam the Netherlands; ^3^ Department of Animal Sciences, Behavioral Ecology Group Wageningen University Wageningen the Netherlands

**Keywords:** Poeciliidae, *Poeciliopsis gracilis*, sperm longevity, sperm retention, viviparity

## Abstract

Sperm storage is a post‐copulatory strategy by which females can improve their fecundity by creating asynchrony between mating and fertilization. Sperm storage duration varies across vertebrate species, wherein longer sperm storage is thought to coincide with better reproductive success. Among the vertebrates, live‐bearing fishes of the family Poeciliidae are generally assumed to store sperm for extended periods of time, but the temporal dynamics of this process remain unknown for most species. To date, research suggests that superfetatious poeciliids—which give birth to more frequent, temporally overlapping broods—may be able to store sperm longer than non‐superfetatious species. However, robust empirical data for superfetatious poeciliids is very limited. Here, we assess the maximum duration of sperm storage and usage in the superfetatious poeciliid *Poeciliopsis gracilis* by comparing offspring production over time for both sexually isolated (single) and paired fish. We found that (a) the majority of *P. gracilis* females can store sperm for a maximum of 5 months with a smaller fraction of individuals able to extend this period to nearly 7 months, likely by “skipping broods,” and (b) the number of offspring produced decreases over time post‐isolation. With this study, we expand our knowledge of post‐copulatory strategies by providing an assessment of both sperm storage longevity and its impact on offspring production over time in a superfetatious, live‐bearing fish from the family Poeciliidae. We aim to encourage further research to generate and publish data on sperm storage longevity across the family Poeciliidae to elucidate how sperm storage varies across species with different reproductive strategies.

## Graphical abstract

Among the vertebrates, live‐bearing fishes of the family Poeciliidae are generally assumed to store sperm for extended periods of time, but the temporal dynamics of this process remain enigmatic for most species. With this study, we assess the maximum duration of sperm storage and usage in the superfetatious poeciliid *Poeciliopsis gracilis* by comparing offspring production over time for both sexually isolated (single) and paired fish. We found that (a) the majority of *P. gracilis* females can store sperm for a maximum of 5 months, with a smaller fraction of individuals able to extend this period to nearly 7 months, likely by “skipping broods” and (b) the number of offspring produced decreases over time post‐isolation.

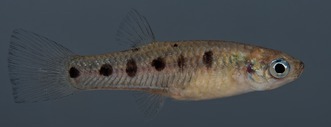

## 1 Introduction

Female sperm storage is a post‐copulatory reproductive strategy by which females temporally decouple the act of mating from the moment of fertilization by internally storing viable spermatozoa for extended periods of time after copulation (Orr and Zuk 2012; Birkhead and Møller 1993; Holt and Lloyd 2010; Holt and Fazeli 2016). Sperm storage has been described in a wide variety of vertebrate species, including reptiles, birds, mammals, and fishes (Birkhead and Møller 1993; Holt and Lloyd 2010; Holt and Fazeli 2016; Neubaum and Wolfner 1998; Howart 1974 & others). The duration of sperm storage varies considerably among species, from a couple of hours (e.g., mammals) to several years (e.g., reptiles & fishes; Birkhead and Møller 1993; Holt and Fazeli 2016). The major evolutionary advantage afforded by storing sperm is the asynchrony created between mating and fertilization, where the temporal separation between these events is restricted by the maximum longevity of the sperm storage. The longer females can store sperm, the more control they have over the reproductive process. Long‐term sperm storage allows for: (1) better synchronicity between fertilization and a female's reproductive cycle, (2) fertilization with stored sperm when males are scarce, and (3) lower copulation frequency when mating is dangerous or deleterious (Birkhead and Møller 1993; Holt and Lloyd 2010). Many species with sperm storage mechanisms are also polyandrous, meaning females have the opportunity to store sperm from multiple males simultaneously, allowing processes like sperm competition and/or cryptic female choice to promote fertilization by specific males (Constantz 1984; Birkhead and Møller 1993). Therefore, storing sperm not only offers females a competitive advantage during post‐copulatory sexual selection but can also directly improve a female's fecundity and overall fitness (Birkhead and Møller 1993; Neubaum and Wolfner 1998).

Live‐bearing fishes from the family Poeciliidae are a prime example of the evolutionary advantages owed to storing sperm: fishes from this family are predominantly polyandrous and the majority of species mate through a process known as coercive mating, whereby males mate non‐consensually with females, reducing their pre‐copulatory sexual selective power (Pilastro et al. 1997; Bisazza and Pilastro 1997; Farr 1989; Evans and Pilastro 2011). To our knowledge, the ability to produce offspring after isolation from males was first reported in poecillids in 1851 (Poey 1851), with the first reports on sperm storage as the mechanism for this reproduction in the early 1900s (Philippi 1908; Winge 1922; Van Oordt 1928; Schmidt 1920). In the last century, a plethora of research has been focused on the localization, morphology, and functionality of these sperm stores (Winge 1937; Constantz 1989; Greven 2011; Uribe et al. 2010; Hankison et al. 2022), wherein researchers have broadly determined that short‐term sperm storage (several days or weeks) is consolidated to the folds of the ovarian epithelium while long‐term sperm storage (several months) is localized in the epithelial folds of the gonoduct (Greven 2011). However, despite poeciliids being a popular fish for both researchers and aquarium hobbyists, it is currently not well known how long sperm can be stored across different poeciliid species.

While it is often claimed that poeciliids can store sperm for several months (Greven 2011; Constantz 1989; Vallowe 1953), there is limited empirical data supporting these claims, with researchers often relying on anecdotal reports or data generated from very few fish, as shown in Table 1. To date, only two papers have empirically determined the maximum duration of sperm storage in Poeciliidae: Vallowe (1953) in *Xiphophorous helleri* and Gasparini and Evans (2018) in *Poecilia reticulata* (Table 1). Vallowe (1953) studied 5 females housed in isolation from males and proceeded to re‐pair their isolated fish with males after “*it was believed that they had produced their last broods*” on stored sperm; therefore, this study did not establish a maximum longevity of sperm storage. Gasparini and Evans (2018), conversely, performed an assessment of 25 females isolated from males after natural or artificial insemination and found that females were capable of storing sperm for a maximum of approximately 6 months, with brood sizes decreasing over time. However, they emphasize that “*this result needs to be interpreted cautiously as fewer than 10 females produced a fourth brood*,” meaning their maximum sperm storage for the majority of individuals was ≈4 months.

**TABLE 1:** An overview of the poeciliid literature, to date, which investigates sperm storage longevity and offspring production over time. References are listed in chronological order from oldest to newest for both non‐superfetatious and superfetatious species.

Species	*n*	Duration of sperm storage	References
*Unknown*	—	> 2 months^a^	Poey (1851)
**Non‐superfetatious**			
*Cnesterodon decemmaculatus*	—	> 2.5 months^b^	Zolotnisky (1901)
*Poecilia reticulata*	1	≥ 7 months^b^	Schmidt (1920)
*Xiphophorous helleri*	7	≥ 10 months^b^	Van Oordt (1928)
*Poecilia reticulata*	1	≈6–8 months^b^	Purser (1937)^c^
*Poecilia reticulata*	1	≈7 months^a^	Winge (1937)^d^
*Xiphophorous helleri*	—	≈7 months^a^	Clark (1950)
*Xiphophorous maculatus*	5	≈4.5 months^b^	Vallowe (1953)^f^
*Poecilia reticulata*	—	8 months^a^	Kadow (1954)
*Belonesox belizanus*	4	> 1.5 months^b^	Turner and Snelson (1984)
*Poecilia reticulata*	25	≈4–6 months	Gasparini and Evans (2018)
**Superfetatious**			
*Heterandria formosa*	—	10 months^a^	Turner (1947)^e^, ^f^
*Poeciliopsis gracilis*	23=	5–7 months	*This study*

^a^ Value is reported without direct experimentation/citation.

^b^ Value is the maximum reported in this article, but experiments were not conducted long enough to determine the actual *maximum* duration of sperm storage for this species.

^c^ Reports 6–8 months pers. comms. but only tests up to ≈4 months by time of publication.

^d^ Reports production of 8 broods post‐isolation, self‐citing Winge (1922, 1927) but neither paper directly studies or reports sperm storage duration.

^e^ Based on sperm found in the genital ovary of isolated females during dissection.

^f^ This is the first paper to report that fish produced decreasing numbers of offspring over time when isolated from males.

**FIGURE 1:**
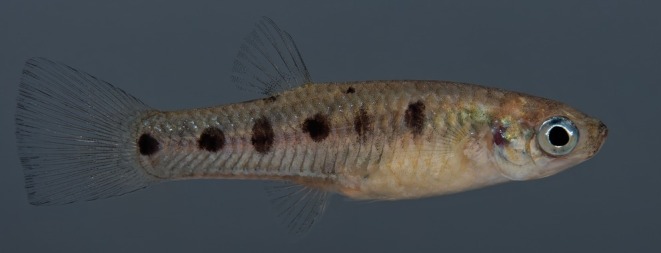
A representative photograph of a *Poeciliopsis gracilis* female. Photo credit: J. L. van Leeuwen.

Here, we study sperm storage longevity in the porthole livebearer, *Poeciliopsis gracilis* (family Poeciliidae; Figure 1), a superfetatious live‐bearing species (Pollux et al. 2009). Many poecillids (≈35%) are also superfetatious, meaning females can carry multiple broods of offspring at different developmental stages simultaneously (Turner 1937; Pollux et al. 2009; Dekker et al. 2022). The genus *Poeciliopsis* is particularly interesting because all its members have superfetation (Pollux et al. 2014). Superfetation is generally characterized by a more frequent production of litters, often in association with smaller litter sizes (Reznick and Miles 1989). Moreover, superfetatious species tend to have more and larger spermathecae (sperm storage organs) compared to non‐superfetatious poeciliid species (Olivera‐Tlahuel et al. 2017). This raises the question whether superfetatious species have a capacity to store sperm for longer periods of time compared to non‐superfetatious species. The current, albeit limited, data (Table 1) tentatively suggest that sperm storage duration might be longest in the only superfetatious species ( *Heterandria formosa* ) studied to date, compared to other non‐superfetatious poeciliid species (Table 1). However, more data is needed to truly investigate this potential relationship between superfetation and sperm storage longevity. With this study, we hope to stimulate future investigations into sperm storage longevity in poeciliid fishes by (1) establishing the maximum sperm storage longevity in a superfetatious live‐bearing species and (2) assessing how offspring production (and sperm utilization) changes over time as sperm stores are depleted.

## 2 Materials and Methods

### 2.1 Birth‐Tracking of Paired and Single *P. gracilis*

All fish used in this study were individuals originally set up in preparation for other experiments. The fish used for the present study originate from three different experimental cohorts (cohorts 1, 2, and 3, respectively; see details below) wherein cohort 1 was tangentially used in another study (Ernst et al. 2024), cohort 3 was surplus individuals from that experiment, and cohort 2 was added afterwards to maintain a robust number of females per treatment for investigating the maximum duration of sperm storage. Cohort numbering (1–3) represents the length of time fish underwent birth‐tracking, with cohort 1 being tracked for the least amount of time and cohort 3 being tracked for the maximum amount of time. *Poeciliopsis gracilis* (Heckel 1848) used in this study were reared in 30 L tanks at 25°C ± 2°C and fed to satiation on a diet of sterilized CAVIAR (BernAqua) and nauplii *Artemia* sp. At the start of the experiment (*t* = 0 days post‐isolation), all male fish were removed from the 30 L tanks. Females were then housed in all‐female group tanks and isolated from male contact for the first 78 (cohorts 1 and 3) or 134 (cohort 2) days. After this period of isolation, females were randomly selected and rehoused in 13 L tanks (Figure S2), where paired fish were housed with a randomly selected male to allow for mating, while single females were housed individually to prevent mating and the acquisition of new sperm. Paired females acted as an additional control for the effects of aging on reproduction, fecundity, and sperm utilization. During this period, henceforth known as the birth‐tracking period, fish were monitored daily for individual offspring production.

Since females were initially housed in all‐female group tanks, their offspring production could, depending on the cohort, only be studied after 78 or 134 days post‐isolation, as described below and shown in Figure S1:

- *Cohort 1*: (paired: 12, single: 12) birth‐tracking took place from 78 to a maximum of 161 days post‐isolation. Fish in this group were additionally used for other experiments (see Appendix S1 & Ernst et al. (2024)).
- *Cohort 2*: (paired: 10, single: 8) birth‐tracking from 134 to 274 days post‐isolation.
- *Cohort 3*: (paired: 6, single: 7) birth‐tracking from 78 to 274 days post‐isolation.

A more detailed description of the methodology (including feeding regimes and tank specifications) is provided in Appendix S1.

### 2.2 Data Analysis

A total of 55 fish were analyzed: 28 paired and 27 single. All data analyses were performed in R version 4.3.2 (R Core Team 2023) in RStudio (Posit Team 2023). All final code and a description of the full analysis are provided in Appendix S3; provided code and analytical protocols can be directly used by other researchers aiming to repeat our study in other poeciliid species. Briefly, the number of offspring per brood and the time between broods for all cohorts were analyzed using a combination of summary statistics and mixed models generated using the *pscl* package (Zeileis et al. 2008). To generate summary statistics, all data were tested for normality using Shapiro‐Wilk normality tests and due to non‐normality, data were analyzed using either a Wilcoxon‐rank sum test or a Kruskal‐Wallis ranked sum test along with pairwise comparisons made with a Dunn test. Before modeling, the days when broods were recorded were converted into a time in days post‐isolation, where the brood day (day *n*) was calculated relative to the day in which fish began sexual isolation (day 0). These relative brood times were then binned into 14 bins of 14 days each, as shown in Figure S3. Two‐week intervals were chosen since this is close to the mean interbrood interval reported for other poeciliid species (Reznick et al. 1996). The number of offspring per brood over time for both single and paired fish was analyzed using a zero‐inflated negative binomial (ZINB) model with the interaction of time post‐isolation (binned days) × status (single or paired females) as the predictor variable for both the Poisson and Bernoulli processes. This same model was used to analyze each cohort individually as well as all the data combined. Due to consistent results across all three cohorts, only the combined results will be discussed. Individual cohort results can be found in Appendix S4 (Figure S4 and Table S1). The time between broods over time for single and paired fish was analyzed using a generalized linear model (GLM; family = Gamma) with the interaction of time post‐isolation (binned days) × status (single or paired) as the predictor variable. Given that single fish had decreasing brood sizes after bin 6, additional analyses were also run on a truncated version of this data which only considers bins 1–6 (Appendix S4, Figure S5a,b and Table S2). We neglected to measure final, female wet mass and/or standard length for all individuals at the end of the experiment and thus this factor was not included in our models. However, by randomly selecting females to be included in both groups (paired and single) and given our large sample sizes, we expect that female size/mass distribution will be relatively homogenous across both groups.

## 3 Results and Discussion

### 3.1 The Number of Offspring per Brood Declines Over Time in Isolated Females

All 28 paired and 23 of 27 single fish gave birth at least once during the birth‐tracking period. The 4 single fish which did not give birth during this period likely (1) were not successfully inseminated during the rearing period, (2) had used up their stored sperm during the sexual isolation period but before the birth‐tracking period, and/or (3) simply did not use their stored sperm. Given that mating attempts in poeciliids are often unsuccessful (Bisazza and Marin 1995) and that our isolation period lasted either 78 or 134 days for single fish (cohorts 1 & 3 or cohort 2)—enough time for fish to produce ≈5–8 broods—either hypothesis is possible. Therefore, we can consider the single group as having a final number of 23 fish who were accurately assessed through our experimental methods.

**FIGURE 2:**
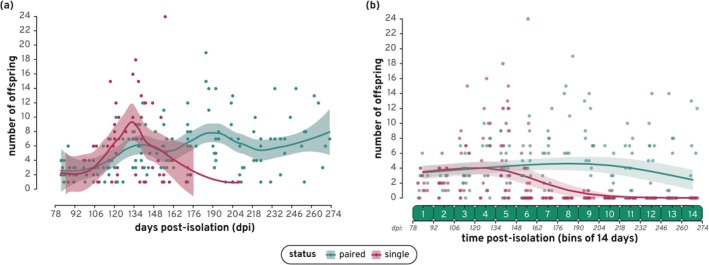
Offspring per brood over time: (a) A scatter‐plot of the number of offspring produced for both paired and single fish over time in days post‐isolation (dpi). Offspring production was not recorded for the first 78 (for cohorts 1 and 3) or 134 (for cohort 2) days post‐isolation. Fitting lines for each group include 95% confidence intervals shown by the transparent ribbons. (b) The number of offspring over time, in binned days post‐isolation, for paired and single fish in all cohorts combined as predicted by our ZINB model. Lines indicate the model predictions of the effect of the interaction variable (status [single/paired] × time post‐isolation [binned days]), with the lower and upper bounds of the 95% confidence interval shown by the transparent ribbons. Points on the graph show the actual number of offspring per fish in each bin used to generate the model.

Single fish had overall fewer cumulative offspring during the birth‐tracking period and a lower average offspring per brood regardless of time (Figure S4a,b and Appendix S3). This is to be expected as single fish predominantly stopped giving birth at or before bin 6 (162 days or 5.3 months post‐isolation) and therefore produced significantly fewer broods overall (*W* = 8038.5, *p* = 5.22e‐09). The number of offspring per brood over time (binned days) for all fish was analyzed individually for each cohort and then for all cohorts combined (Figure S4c; Appendix S3). Overall, single fish had a decreasing number of offspring per brood over time, particularly after bin 6 (Figure 2a). This establishes that the majority (19/23) of *P. gracilis* females exhausted their sperm stores after ≈5 months. Figure 2a shows that while single fish increased offspring production drastically at the start of the birth‐tracking period, this production quickly declined, with the last single fish (S26) producing only 1 offspring in bin 10. Meanwhile, paired fish increased offspring production more slowly and continued to fluctuate around the mean up until the end of the birth‐tracking period.

After analyzing all cohorts in separate ZINB models (Figure S4c and Appendix S3), we combined all the cohorts together to determine if status (single or paired) had an impact on the number of offspring per brood over time (number of days post‐isolation) for all fish in the experiment. While both single and paired fish began the birth‐tracking period producing 3 to 4 offspring per brood (paired: 3.4 ± 0.45 [SE]; single: 3.5 ± 0.41 [SE]), single fish ended the birth tracking period producing 0 (0.01 ± 0.03 [SE]) offspring per brood while paired fish continued to produce approximately 2 (2.4 ± 0.64 [SE]) offspring per brood. Additionally, the effect of time (days post‐isolation) had a lower *p*‐value for paired fish than for single fish, most likely due to the increasing chance for these fish of producing zero offspring over time (Table 2a). This zero‐offspring frequency increased over time for both groups, meaning that fish are more likely to stop producing offspring near the end of the birth‐tracking period, but single fish have a higher zero‐offspring frequency over time compared to paired fish. These findings align with the observations of both Vallowe (1953) and Gasparini and Evans (2018) who found that female poeciliids produced less offspring over time post‐isolation and primarily stopped producing offspring after ≈4 months. Similar to these studies, we assess sperm storage by looking at offspring production over time without directly investigating the quantity of stored sperm or its localization. Therefore, future studies should investigate whether females stop producing offspring due to a depletion in sperm stores or a lack of viable stored sperm (i.e., due to sperm death).

**TABLE 2:** Mixed model predictions: (a) Outcomes from the ZINB model for the number of offspring per brood for all cohorts combined. Time post‐isolation is abbreviated as tpi. (b) Outcomes from the GLM model for the time between broods for all cohorts combined. Significance is marked with stars where: *p < 0.05 and ***p < 0.001.

	Estimate	SE	*z*	*p*
*(a) Offspring per brood*				
Count model coefficients: intercept	1.17	0.14	8.24	*p <* 0.001***
tpi × status (paired)	0.06	0.02	3.52	*p <* 0.001***
tpi × status (single)	0.20	0.04	2.52	*p <* 0.05*
log(theta)	0.71	0.12	3.83	*p <* 0.001***
Zero‐inflation model coefficients: intercept	−5.17	0.85	−6.05	*p <* 0.001***
tpi × status (paired)	0.43	0.07	5.84	*p <* 0.001***
tpi × status (single)	0.86	0.13	6.53	*p <* 0.001***
*(b) Time between broods*				
Intercept	0.06	0.004	15.81	*p <* 0.001***
tpi × status (paired)	−0.0004	0.0005	−0.85	0.40
tpi × status (single)	−0.003	0.0008	−3.66	*p <* 0.001***

### 3.2 The Interbrood Interval Increases Over Time for Isolated Females

**FIGURE 3:**
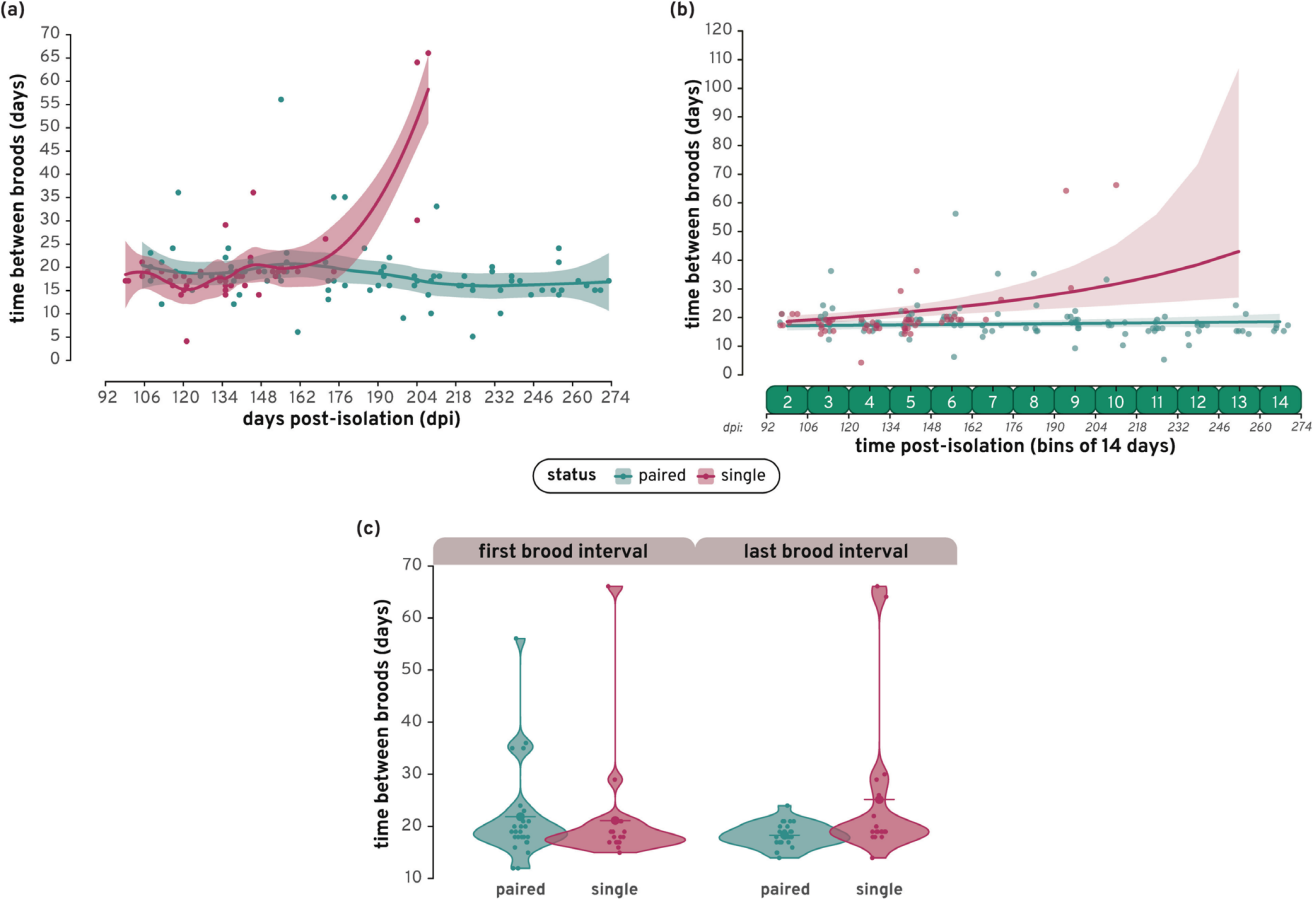
Time between broods (i.e., interbrood interval) over time: (a) A scatter plot of the number of days between broods over time in days post‐isolation (dpi) for paired and single fish. Offspring production, and hence interbrood interval, was not recorded for the first 78 (cohorts 1 and 3) or 134 (cohort 2) days post‐isolation. Fitting lines for each group include 95% confidence intervals shown by the transparent ribbons. (b) The time between broods in binned days post‐isolation, for paired and single fish as predicted by our GLM model. Lines indicate the model predictions of the effect of the interaction variable (status [single/paired] × time post‐isolation [binned days]), with the lower and upper bounds of the 95% confidence interval shown by the transparent ribbons. Points on the graph show the actual time in days between broods per fish in each bin used to generate the model. (c) The number of days between broods for paired and single fish for both their first and last brood intervals. Individual points show the number of days between broods per fish used to generate the violins. Large points with horizontal lines indicate the mean per group.

To determine if time post‐isolation had an impact on the time (in days) between broods, we calculated the time interval between broods (often referred to as the “interbrood interval” in poeciliid research) for all single and paired fish which gave birth to at least 2 broods during the birth‐tracking period (paired: 27, single: 19). Figure 3a shows the raw data, combined for all three cohorts, for both single and paired fish showing that on average, females had a 18.84 ± 7.57 (SD) day interval between each brood, regardless of group. However, given that most single fish stopped producing offspring after bin 6 (162 days post‐isolation), the trend line for single fish is pulled sharply upward by long interbrood intervals from only ≈3/19 single fish (further discussed as “skipped broods” in the following section). Interbrood interval (IBI) varies considerably between poeciliid species, especially between superfetatious and non‐superfetatious species, and can be significantly impacted by environmental factors such as temperature and food availability; in general, superfetatious species like *P. gracilis* have shorter interbrood intervals (12 ± 2 [SE] days) than non‐superfetatious species (32 ± 2.4 [SE] days; Reznick and Miles (1989)). Therefore, an 18.84 ± 7.57 (SD) day IBI is within the normal range for a poeciliid fish like *P. gracilis* .

The number of days post‐isolation significantly influences the interbrood interval in single fish, but not in paired fish (Table 2b; Figure 3a). The time between broods for single fish near the end of the birth‐tracking period (bins 7–14) appears heavily impacted by the increasing interbrood intervals of the 3 of 19 single individuals who produce offspring after brood 6 and then seem to “skip a brood” (see section below and Figure 3c). Here, we characterize “brood‐skipping” as when a fish exhibits a time between broods of equal to or greater than two times the average interbrood interval. When we truncated the data to only include bins 1–6, single fish no longer had a significant difference over time, further suggesting that the trend is heavily impacted by the increasing interbrood intervals of the 3 of 19 single individuals (Figure S5 and Table S2; Appendix S3). These data suggest that across our population of *P. gracilis* females, there might be differing strategies for sperm storage utilization: (1) The predominant strategy in which females slowly but regularly deplete their sperm stores over time post‐isolation or (2) the minority strategy in which some females are able to extend the longevity of their sperm stores by skipping broods. Research has found that superfetaceous poeciliids like *P. gracilis* have more and larger spermathecae (intraovarian structures which enclose and store spermatozoa; Olivera‐Tlahuel et al. 2017), suggesting an evolutionary relationship between superfetation and a large sperm storage capacity.

### 3.3 Sperm Storage Longevity Is Increased for Individuals Which Appear to “Skip Broods”

Figure 3c compares the average first interbrood interval for single vs. paired females with the average last interbrood interval. Here, there is no significant difference between the groups, both within each interbrood interval and between the different time points ($\chi^{2}$ = 6.61, df = 3, *p*‐value = 0.09). Therefore, fish started and ended the birth‐tracking period with the same average interbrood interval, regardless of group. Figure 3c also illustrates the occurrence of brood skipping in our study population. In the first interbrood interval, 4 of 27 paired fish exhibit skipped or late broods (interbrood intervals ≥ 32 days, or 2× the SE of the average interbrood interval).

In the last interbrood interval, all paired fish exhibit a normal interbrood interval, while 2 of 19 (including S26) single fish experience skipped broods. Skipped broods have been scarcely reported in poeciliid literature, and when reported are either attributed to senescence (Reznick et al. 2006) or intraovarian cannibalism (due to stress; Meffe and Vrijenhoek (1981)). When observed and reported as part of analyses on interbrood intervals, skipped broods are considered statistical outliers and are therefore excluded from the final analyses to meet normality requirements for statistical testing (Reznick et al. 1993; Reznick and Endler 1982). It is currently unclear whether the observed incidences of brood skipping in a few single *P. gracilis* females are due to senescence, intraovarian cannibalism or perhaps some other reason. One could argue that the ability to adaptively regulate sperm storage utilization would give females an advantage in highly variable aquatic ecosystems. However, since the incidence of these skipped broods appears to be present in only a small proportion of the overall population, a larger sample size of individuals would be needed to study these different hypotheses. Additionally, assessing the incidence of brood‐skipping across multiple species could provide further insight into the mechanisms that underlie the phenomenon of brood‐skipping and to what extent it is an adaptive strategy for poeciliid females.

## 4 Conclusions and Perspectives

The aim of this study was to determine the maximum longevity of sperm storage in the superfetatious fish *Poeciliopsis gracilis* (family Poeciliidae) and assess how the number of offspring per brood changed with time post‐isolation. Our data indicate that the majority of *P. gracilis* females can retain sperm for ≈5 months, with a subset of individuals able to extend this duration to a maximum of ≈7 months (208 days; fish S26) by “skipping broods.” Over time, as sperm stores deplete, the number of offspring per brood also declines, while the time between broods remains the same, except for the subset of fish who skipped broods. While ≈5 months of sperm storage is not particularly long compared to other vertebrate species (Birkhead and Møller 1993; Holt and Lloyd 2010; Holt and Fazeli 2016), it does represent a significant proportion of the reproductive lifespan for *P. gracilis* : if we assume *P. gracilis* shares a similar reproductive lifespan of ≈800 days with the Guppy ( *P. reticulata* ) then sperm could be retained after copulation for ≈19% of their reproductive lifespan (Reznick et al. 2006). We strongly recommend that future studies (1) house females individually immediately after sexual isolation, (2) track individual offspring production from *t* = 1 days post‐isolation, and (3) record female final wet mass/standard length to account for differences in offspring production related to female size. Additionally, future studies can consider using histology to assess the presence of sperm in the ovary or reproductive tract at the end of the birth‐tracking period as an additional check for unutilized sperm (Olivera‐Tlahuel et al. 2017; Uribe et al. 2016), however, this requires euthanization of the fish and thus prevents their potential usage in future experiments. If sperm storage longevity data were to be systematically collected and reported across the family Poeciliidae, future research could investigate (1) whether sperm storage duration is impacted by a species reproductive strategy (e.g., superfetation, lecithotrophy, matrotrophy, clonality), (2) to what extent sperm storage and sperm utilization dynamics may have contributed to the evolutionary transition from non‐superfetatious to superfetatious, and (3) the frequency of “brood‐skipping” across different species and to what extent this is an adaptive strategy controlled by females or a passive result of depleting sperm stores. By answering these questions, researchers would not only have more information about sperm storage utilization to inform and plan their experiments, but we would also have better insights into the poeciliid female's post‐copulatory, reproductive power.

Previous research in other poeciliid fishes (summarized in Table 1), shows that sperm storage generally lasts anywhere from 2 to 10 months (≈60–304 days) post‐isolation, with the only superfetatious species, *Heterandria formosa*, retaining sperm for the maximal 10 months. Our finding that the superfetatious *P. gracilis* can store sperm for a maximum of 5–7 months does not support the idea that superfetatious species have a longer maximum sperm storage duration compared to non‐superfetatious poeciliid species. This study reports an experimentally derived maximum duration of sperm storage in a superfetatious poeciliid fish and should be followed by further investigation in other poeciliids to determine how sperm storage longevity may change across species with different reproductive strategies.

## Author Contributions

**T. R. Ernst:** conceptualization (equal), data curation (lead), formal analysis (lead), investigation (lead), methodology (lead), project administration (lead), visualization (lead), writing – original draft (lead). **R. M. H. W. Hogers:** data curation (equal), investigation (equal), methodology (equal), writing – review and editing (supporting). **P. J. C. Kwant:** data curation (supporting), writing – review and editing (supporting). **A. Korosi:** conceptualization (equal), project administration (supporting), supervision (equal), writing – review and editing (supporting). **J. L. van Leeuwen:** conceptualization (equal), methodology (supporting), project administration (supporting), supervision (equal), writing – review and editing (supporting). **A. Kotrschal:** conceptualization (equal), formal analysis (supporting), project administration (supporting), supervision (equal), writing – review and editing (equal). **B. J. A. Pollux:** conceptualization (equal), funding acquisition (equal), project administration (equal), resources (equal), supervision (equal), writing – review and editing (equal).

## Conflicts of Interest

The authors declare no conflicts of interest.

## Supporting information


**Appendix S1:** ece372086‐sup‐0001‐Appendixs.docx.

**Figure S1:** An overview of all the paired and single fish studied in this experiment. All fish received a high‐food diet during the birth‐tracking period except for during the normal‐food diet indicated by the blue boxes or during the behavioral testing indicated by the brown boxes. Fish represented by faded bars reached a humane endpoint prior to the end of the experiment and are excluded from analysis.

**Figure S2:** A schematic of the tanks used to house fish during the birth‐tracking period. (a) A 3D depiction of the entire 6‐compartment tank, indicating that fish can see fish in neighboring tanks but cannot physically interact with them. (b) A top‐down view of one of the home compartments where fish were housed.

**Figure S3:** An overview of the binned days post‐isolation used for all statistical analysis. Day 78 post‐isolation is the first day that fish begin birth‐tracking and fish continue in this period until the end of the experiment at 274 days post‐isolation.

**Figure S4:** Offspring per brood per cohort: (a) The cumulative number of offspring produced by individual single and paired fish during the birth‐tracking period per cohort: (i) cohort 1, (ii) cohort 2, and (iii) cohort 3. Cumulative sum lines are colored to indicate status (paired or single) and end with a point to indicate the final cumulative sum for each individual fish. (b) The average number of offspring per brood produced by single and paired fish during the birth‐tracking period per cohort: (i) cohort 1, (ii) cohort 2, and (iii) cohort 3. Individual points show the number of offspring per brood data used to generate the violins. The large points with horizontal lines indicate the mean per group. Significance between groups is marked with a star. (c) The number of offspring over time, in binned days post‐isolation, for paired and single fish in each of our cohorts as predicted by our ZINB models, where: (i) cohort 1, (ii) cohort 2, and (iii) cohort 3. Lines indicate the model predictions of the effect of the interaction variable (status (single/paired) × time post‐isolation (binned days)), with the lower and upper bounds of the confidence interval shown by the transparent ribbons. Points on the graphs show the actual number of offspring per fish in each bin used to generate the model.

**Figure S5:** Time between broods (i.e., interbrood interval) over time for bins 1–6: (a) A scatter‐plot of the number of days between broods over time for paired and single fish during bins 1–6. Fitting lines for each group include 95% confidence intervals shown by the transparent ribbons. (b) The time between broods in days post‐isolation, for paired and single fish as predicted by our ZINB model, where only the first 6 bins were analyzed. Lines indicate the model predictions of the effect of the interaction variable (status (single/paired) × time post‐isolation (binned days)), with the lower and upper bounds of the 95% confidence intervals shown by the transparent ribbons. Points on the graph show the actual time in days between broods per fish in each bin used to generate the model.

**Table S1:** Outcomes from the ZINB models for the number of offspring per brood for each cohort and for all data combined. Time post‐isolation is abbreviated as tpi. Significance is marked with stars where: *p < 0.05, **p < 0.01, and ***p < 0.001.

**Table S2:** Outcomes from the GLM model for the time between broods in bins 1–6 for all cohorts combined. Time post‐isolation is abbreviated as tpi. Significance is marked with stars where: *p < 0.05 and ***p < 0.001.

## Data Availability

The data, code, and analyses that support the findings of this study are available in Figshare in: Appendix S2 (data; 10.6084/m9.figshare.24032028) and Appendix S3 (code & analyses; 10.6084/m9.figshare.24032109).
