# Supplementary material for: Long‐Term Sperm Storage in a Superfetatious Live‐Bearing Fish (Poeciliopsis gracilis, Poeciliidae)
Source: Ecol Evol. 2025 Sep 4;15(9):e72086. doi: 10.1002/ece3.72086 (PMC12410990; doi:10.1002/ece3.72086)
Supplement: Supplementary file 1 — Appendix S1: ece372086‐sup‐0001‐Appendixs.docx. [file ECE3-15-e72086-s007.docx]

**Appendix S1 – Extended Methods:** Long-term sperm storage in a superfetatious live-bearing fish (*Poeciliopsis gracilis*, Poeciliidae)

T.R. Ernst, R.M.H.W. Hogers, P.J.C. Kwant, A. Korosi, J.L. van Leeuwen, A. Kotrschal, & B.J.A.

Pollux

## Experimental Animals

All fish used in this study were individuals originally set up in preparation for other experiments. The fish used for the present study originate from three different experimental cohorts (cohorts 1, 2, and 3, respectively; see details below) wherein cohort 1 was tangentially used in another study (Ernst et al., 2024), cohort 3 was surplus individuals from that experiment, and cohort 2 was added afterwards to maintain a robust number of females per treatment for investigating the maximum duration of sperm storage. Cohort numbering (1–3) represents the length of time fish underwent birth-tracking, with cohort 1 being tracked for the least amount of time and cohort 3 being tracked for the maximum amount of time. *Poeciliopsis gracilis* (Heckel, 1848) used in this study were reared in 30 L tanks (density ≈ 1 fish/L) with constant aeration, water filtration via recirculating bio-filter and a water temperature of 25 ± 2° C under a 10 hr light/ 14 hr dark cycle in the Live-Bearing Fish Facility of CARUS (Wageningen University & Research; Wageningen, The Netherlands). Fish were fed to satiation on a diet of 0.15-0.2 grams of 200–300 µm sterilized CAVIAR (BernAqua) and ≈ 8 mg nauplii *Artemia spec.* (Salt Lake Aquafeed, Premium Artemia Cysts, hatched on-site in CARUS) daily at 8:00 and 16:00, respectively. All the *P. gracilis* used in this study were used under approval by the animal ethics committee of Wageningen University & Research (2020.W-0027).

Fish were allowed to breed freely after sexual maturation with any number of males available in their tank. At the start of the experiment (t = 0 days post-isolation), all male fish were removed from the 30 L tanks, during which offspring were collected from the tanks when necessary. Females were then housed in all-female group tanks and isolated from male contact for the first 78 (cohorts 1 & 3) or 134 (cohort 2) days. After this period of isolation, 45 females (paired: 20, single: 25) were randomly selected and rehoused in 13 L tanks (Figure S2), where paired fish were housed with a randomly selected male to allow for mating, while single females were housed individually to prevent mating and the acquisition of new sperm. These 45 females were originally set up in preparation for behavioral testing (as described in Ernst et al. (2024)), but were later randomly sub-divided into cohort 1 which was used for behavioral testing and cohort 3 which were surplus individuals. After 134 days post-isolation, 18 females (paired: 10, single: 8) from the rearing tanks were randomly selected and rehoused in the same system of 13 L tanks; these fish became cohort 2.

Each tank consisted of a 14 × 37 × 29 (width × depth × height) cm, 13 L compartment within a larger 6-compartment tank, as shown in Supplemental Figure S2. The compartment structure of the tanks allowed fish to see and interact with at least one neighboring fish (fish housed in compartments on the end of each tank had access to only one neighbor), to reduce isolation stress, particularly in single females. Each tank also contained a plastic aquarium plant for fish to use as shelter and enrichment. Fish in these 13 L tanks were fed a high-food diet, where they received an additional meal on weekdays (at ≈ 12:00) to promote offspring production: *ad libitum* adult *Artemia* mixed with garlic (Aquadip #0355; defrosted in tank water prior to administration). This high-food diet was temporarily reduced to a normal-food diet (no additional meal on weekdays) for a 1.4 month period directly following the behavioral experiments (161–203 days post-isolation) due to reduced staffing during the winter holidays. The high-food diet was re-instated after this period.

## Birth-Tracking

Once moved to their individual tanks, all fish were monitored daily for individual offspring production. If offspring were found in the tank on weekdays, the date and number of offspring were recorded before the offspring were removed and rehoused for rearing. On weekends, only the date was recorded, and the number of offspring was recorded on the following Monday when the offspring were removed from the tank and rehoused. Since females were initially housed in all-female group tanks, their offspring production could, depending on the cohort, only be studied after 78 or 134 days post-isolation, as described below and shown in Figure S1:

- **cohort 1:** (paired: 12, single: 12) fish began the birth-tracking period at 78 days post-isolation and underwent a behavioral experiment (Ernst et al., 2024) starting at 119 days post-isolation. These fish each ended their birth-tracking on a different day depending on when they finished behavioral testing, with the last fish finishing birth-tracking at 161 days post-isolation.
- **cohort 2:** (paired: 10, single: 8) fish began the birth-tracking period at 134 days post-isolation and continued to be tracking until the end of the experiment at 274 days post-isolation.
- **cohort 3:** (paired: 6, single: 7) fish began the birth-tracking period at 78 days post-isolation and continued to be tracked until the end of the experiment at 274 days post-isolation.

## Data analysis

Data were collected from 63 fish in total: 30 paired and 33 single fish. However, 8 fish (paired: 2, single: 6) were omitted from the analysis because they reached a humane endpoint prior to the end of the birth-tracking period. Humane endpoint is reached when fish show signs of illness or injury which are detrimental to their welfare and thereby participation in the given study. Therefore, a total of 55 fish were analyzed: 28 paired and 27 single. All data analyses were performed in R version 4.3.2 (R Core Team, 2023) in RStudio (Posit team, 2023). For a full description of the data analysis and associated code see [Appendix S3.](https://figshare.com/s/0a5919fa08a77d6f6e17)

# APPENDICES S2 & S3 – DATA & ANALYSES: Long-term sperm storage in a superfetatious live-bearing fish (Poeciliopsis gracilis, Poeciliidae)

[Appendix S2](https://figshare.com/s/b4ac6849d6ca4fb5cc33) contains all of the data presented and analyzed in this study. A README file, which fully describes the metadata for all data files, is included when downloading the data. [Appendix S3](https://figshare.com/s/0a5919fa08a77d6f6e17) contains an interactive html file which provides all of the R code used to perform the analyses and generate the plots presented in this study. These appendices are temporarily available via a private link at Figshare which can be accessed until 31/12/2037 by scanning the QR code below. If/when the study is published, the private link will be deactivated and these appendices will become publicly available through Figshare.


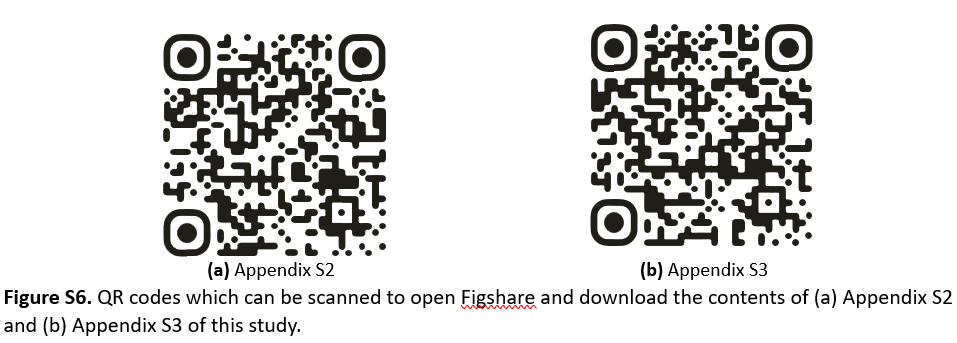


# APPENDIX S4 – EXTENDED RESULTS: Long-term sperm storage in a superfetatious live-bearing fish (Poeciliopsis gracilis, Poeciliidae)

T.R. Ernst, R.M.H.W. Hogers, P.J.C. Kwant, A. Korosi, J.L. van Leeuwen, A. Kotrschal, & B.J.A.

Pollux

## Number of offspring per brood per cohort

### Cohort 1

Fish from the cohort 1 were birth-tracked for a maximum of 83 days or 2.7 months (bins 1–6) during which they were trained and tested in a cognitive task (Ernst et al., 2024) in the latter 42 days (bins 4–6). Supplemental Figure S4a(i) shows the cumulative sum of offspring per brood over the binned days post-isolation for each individual fish. The majority of fish from both groups (paired: 8/12, single: 11/12)) give birth up until the end of the birth-tracking period in bins 5 or 6. One fish (S21) had zero offspring during the entire period. Contrary to the other cohorts, single fish had a 2× greater maximum cumulative offspring number, with the maximum cumulative offspring number for a single fish (S19) at 59 offspring compared to 25 offspring for the most productive paired fish (P19). However, overall there was no statistically significant difference in the mean number of offspring per brood between single and paired fish (W = 661.5, p-value = 0.14) when disregarding the timing of the brood (see Supplemental Figure S4b(i)).

To check if status (single or paired) had an impact on the number of offspring per brood over time in the cohort 1, we generated ZINB model predictions for each group spanning from bins 1 to 6, as shown in Supplemental Figure S4c(i). We plotted data from bins 1–14 to reflect the timing of the data relative to the beginning of the birth-tracking period. According to our model, both single and paired fish had a statistically significant increase in the number of offspring per brood over time (Supplemental Table S1a). Both groups begin the birth-tracking period producing ≈ 2 offspring per brood (paired: 2.2 ± 0.41 (SE); single: 2.5 ± 0.45 (SE)). However, single fish end the birth-tracking period producing 5 offspring per brood (4.8 ± 1.98 (SE)), roughly twice the 2 (1.4 ± 0.73 (SE)) offspring per brood produced by paired fish, although with high variation between individuals. The zero frequency — which occur when fish have no offspring in a particular bin and in subsequent bins — increases significantly for both groups over time, with paired fish having a higher zero-frequency compared to single fish (see zero-inflation model coefficients for time post-isolation (binned days) × status; Supplemental Table S1a).

### Cohort 2

Fish from the cohort 2 were in the birth-tracking period for 140 days or 4.6 months (bins 5–14). Supplemental Figure S4a(ii) shows the cumulative sum of offspring per brood over the binned days post-isolation for each individual fish. Here, the majority (6/8) of single fish stop producing offspring before the end of bin 7 (max. 176 days post-isolation) with two fish (S32 & S33) producing 0 offspring and three fish (S28, S29, & S30) producing only 1 offspring by the end of the birth-tracking period. Two single fish, S26 and S27, produced a single offspring per brood in bins 10 and 9 respectively (similar to fish S22 from the cohort 3, see below). Meanwhile, all (n = 10) paired fish continued to produce offspring after bin 7, with 5/10 producing offspring up until the end of the experiment (bin 14). Additionally, paired fish had an approximately 15-fold higher maximum cumulative sum of offspring compared to single fish, with the paired fish P21 producing 77 offspring and the single fish S27 producing only 5 offspring during the birth-tracking period. Over the tracking period, single fish had a significantly lower mean number of offspring per brood compared to paired fish (W = 432.5, p-value = 0.0002; Supplemental Figure S4b(ii)).

To check if status (single or paired) affected the number of offspring per brood over time in the cohort 2, we generated ZINB model-fit predictions for each group spanning from bins 5 to 14 (Supplemental Figure S4c(ii)). Data were plot from bins 1 to 14 to reflect the timing of the data relative to the beginning of the birth-tracking period. According to our model, single fish significantly decreased their number of offspring per brood (Supplemental Table S1b), beginning the birth-tracking period producing less than 1 offspring per brood (0.93 ± 0.45 (SE)) and ending the period producing nearly 0 offspring per bin (0.001 ± 0.02 (SE)). Meanwhile, paired fish did not significantly change their number of offspring per brood, beginning and ending the birth-tracking period producing approximately 3–4 offspring per brood (beginning: 4.8 ± 0.94 (SE); ending: 2.9 ± 0.93 (SE)). As with the cohort 1, the frequency of zeros increased significantly over time for both single and paired fish, although paired fish had a lower zero frequency than single fish (see zero-inflation model coefficients for time post-isolation (binned days) × status; Supplemental Table S1b).

### Cohort 3

Fish from the cohort 3 had the longest period of time in the birth-tracking period, with 196 days (approx. 6.4 months) of birth-tracking (bins 1–14). Supplemental Figure S4a(iii) shows the cumulative sum of the offspring per brood over the binned days post-isolation for each individual fish. The majority of single fish (6/7) stopped having offspring at or before bin 7 (max. 176 days or 5.7 months post-isolation) whereas one fish, S22, gave birth to a single offspring in bin 9, at 204 days (6.7 months) post-isolation (Supplemental Figure S4a(iii)). Meanwhile, all of the paired fish continued to produce offspring after bin 6, with one fish (P14) giving birth to 85 total offspring during the birth-tracking period: more than twice the maximum cumulative sum recorded for single fish (S5: 32 offspring; Supplemental Figure S4a(iii)). During the tracking period, single fish had a significantly lower mean number of offspring per brood compared to paired fish (W = 751.5, p-value = 0.003; Supplemental Figure S4b(iii)).

To determine if status (single or paired) affected the number of offspring per brood over time, we generated ZINB model predictions for each group (Supplemental Figure S4c(iii)), spanning from bins 1 to 14. According to our model, both single and paired fish start the birth-tracking period producing approximately 3 offspring per brood (paired: 3.6 ± 0.62 (SE), single: 3.3 ± 0.53 (SE)) in bin 1. Over time, paired fish significantly decreased (Supplemental Table S1c) their offspring production, producing approximately 1 offspring per brood (1.3 ± 0.74 (SE)) by the end of the experiment (bin 14). Single fish, conversely, do not differ significantly over time (Supplemental Table S1c) although their predicted offspring per brood drops to nearly 0 (0.0006 ± 0.02 (SE)) by the end of the birth-tracking period. Our model also clearly aligns with the raw data, showing a sharp drop in offspring production for single fish after bin 6, with the model predicting nearly 0 offspring for single fish in bins 10–14. The frequency of zeros increases significantly over time for both groups, with single fish having a nearly 2× greater positive impact on the zero frequency over time compared to paired fish (see zero-inflation model coefficients for time post-isolation (binned days) × status; Supplemental Table S1c).

## Time Between Broods or interbrood interval for bins 1–6

Since most single fish stopped giving birth after bin 6, we decided to isolate bins 1–6 into a truncated data set for the time between broods data. Supplemental Figure S5a shows the raw time between broods data for both single and paired fish in bins 1–6 with *loess* (local regression; a non-parametric approach that fits multiple regressions in a local neighborhood) fitting lines to visualize the overall trend of the data. Here it is clear that both single and paired fish have brood intervals fluctuating around 18 days for the duration of the 6 bins. To determine if status (single or paired) had an impact on the time between broods over time for only bins 1–6, we generated GLM model predictions for both groups assuming a Gamma distribution (Supplemental Figure S5b). According to our model, neither single nor paired fish had a significant change in their time between broods over time with both groups beginning the birth tracking period in bin 1 with brood intervals of ≈ 18 days (paired: 18.06 ± 0.004 (SE); single: 17.5 ± 0.004 (SE)) and ending in bin 6 with similar brood intervals (paired: 20.3 ± 0.003 (SE); single: 18.42 ± 0.004 (SE)).
