# Supplementary figures and images for: Long‐Term Sperm Storage in a Superfetatious Live‐Bearing Fish (Poeciliopsis gracilis, Poeciliidae)
Source: Ecol Evol. 2025 Sep 4;15(9):e72086. doi: 10.1002/ece3.72086 (PMC12410990; doi:10.1002/ece3.72086)

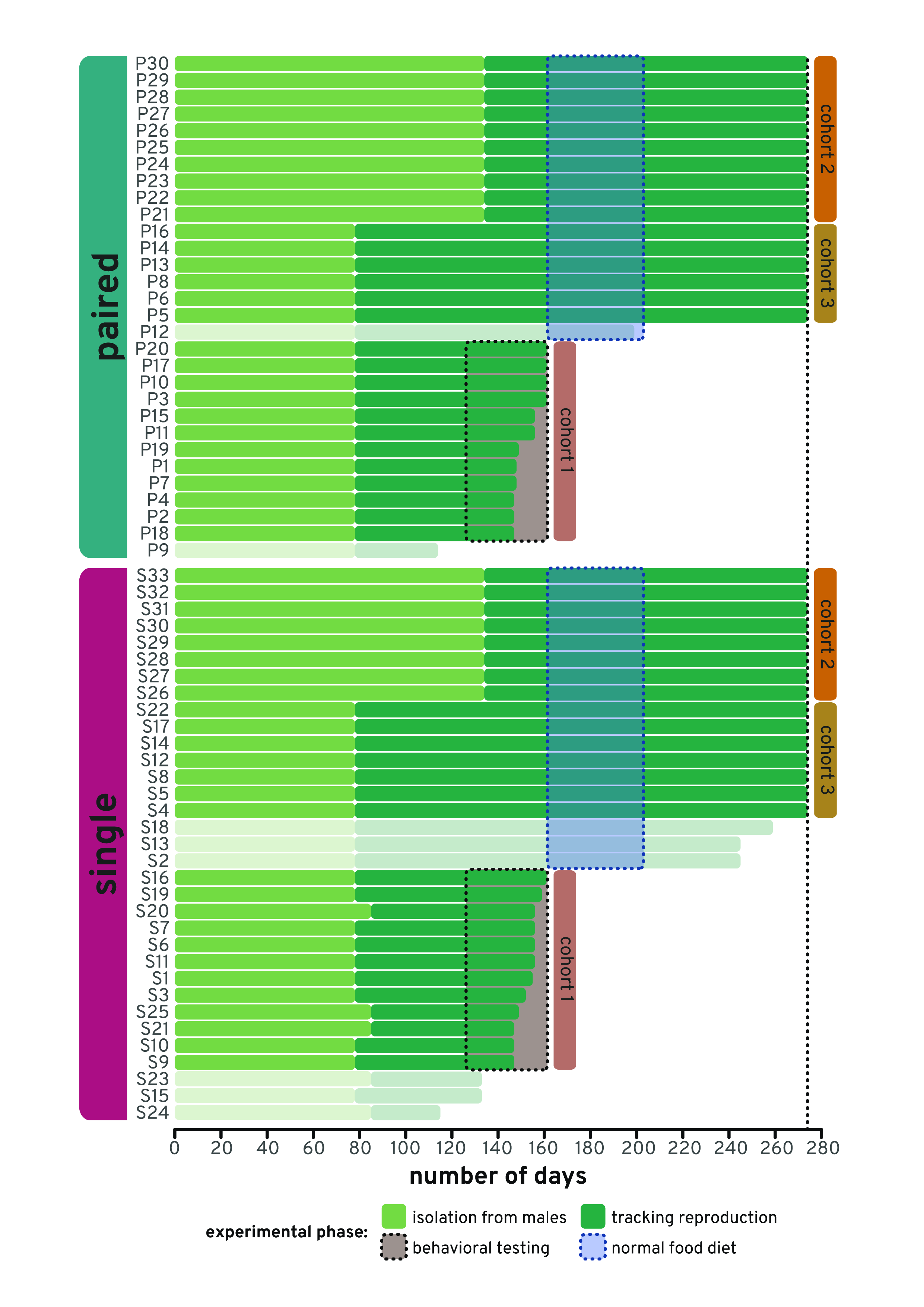

Supplement: Supplementary file 2 — Figure S1: An overview of all the paired and single fish studied in this experiment. All fish received a high‐food diet during the birth‐tracking period except for during the normal‐food diet indicated by the blue boxes or during the behavioral testing indicated by the brown boxes. Fish represented by faded bars reached a humane endpoint prior to the end of the experiment and are excluded from analysis. [file ECE3-15-e72086-s006.tif]

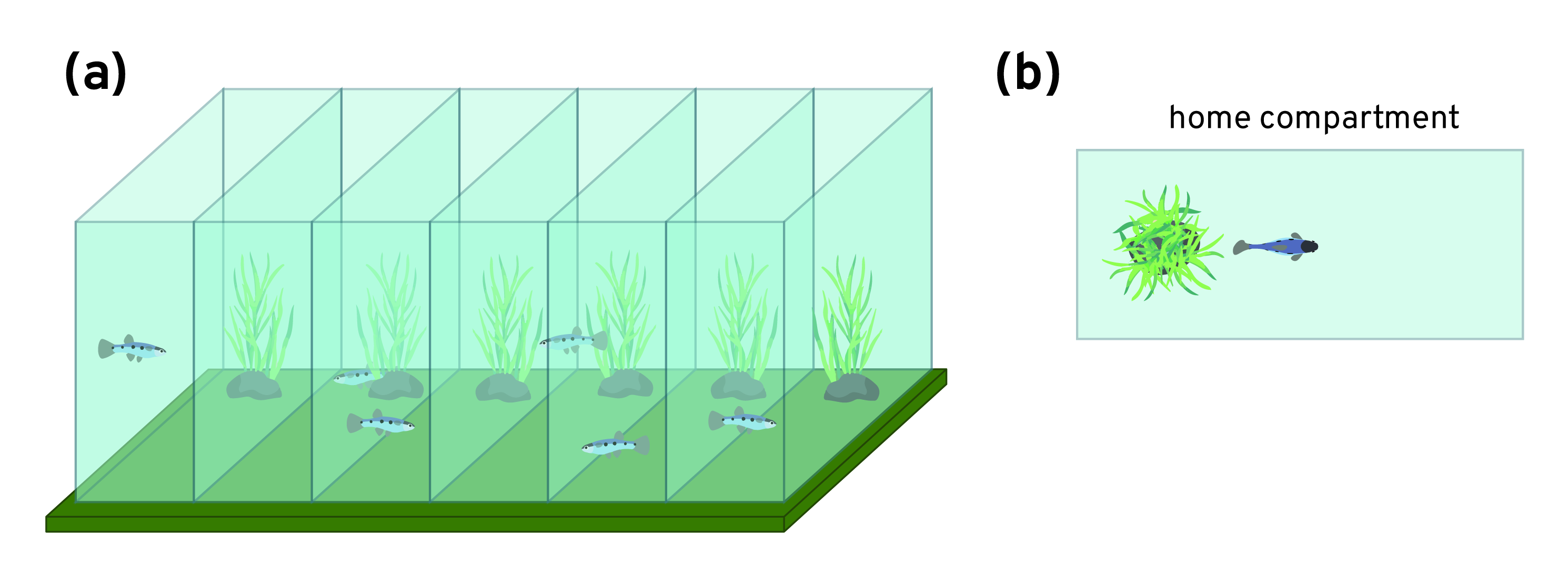

Supplement: Supplementary file 3 — Figure S2: A schematic of the tanks used to house fish during the birth‐tracking period. (a) A 3D depiction of the entire 6‐compartment tank, indicating that fish can see fish in neighboring tanks but cannot physically interact with them. (b) A top‐down view of one of the home compartments where fish were housed. [file ECE3-15-e72086-s001.tif]

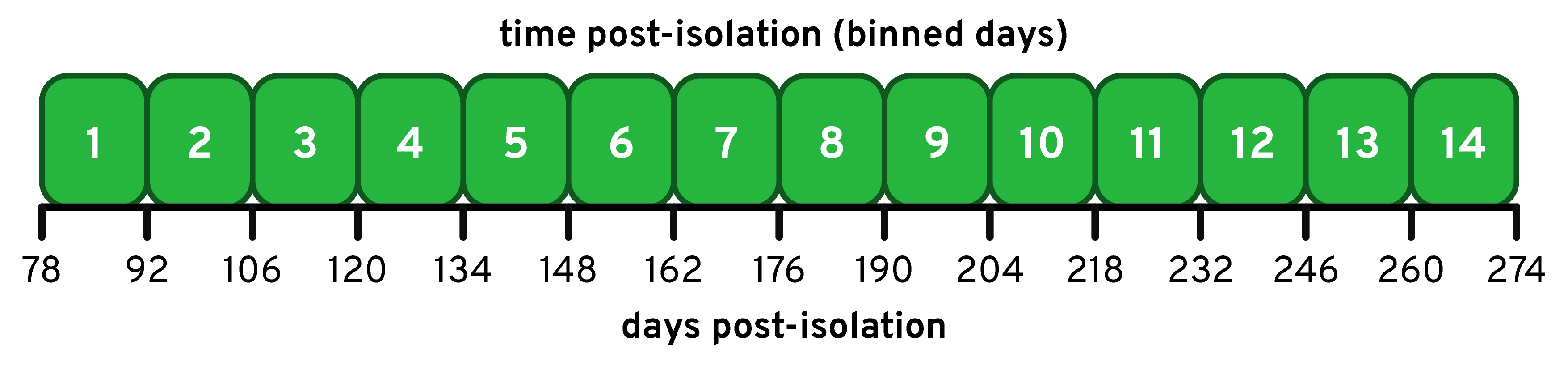

Supplement: Supplementary file 4 — Figure S3: An overview of the binned days post‐isolation used for all statistical analysis. Day 78 post‐isolation is the first day that fish begin birth‐tracking and fish continue in this period until the end of the experiment at 274 days post‐isolation. [file ECE3-15-e72086-s003.tif]

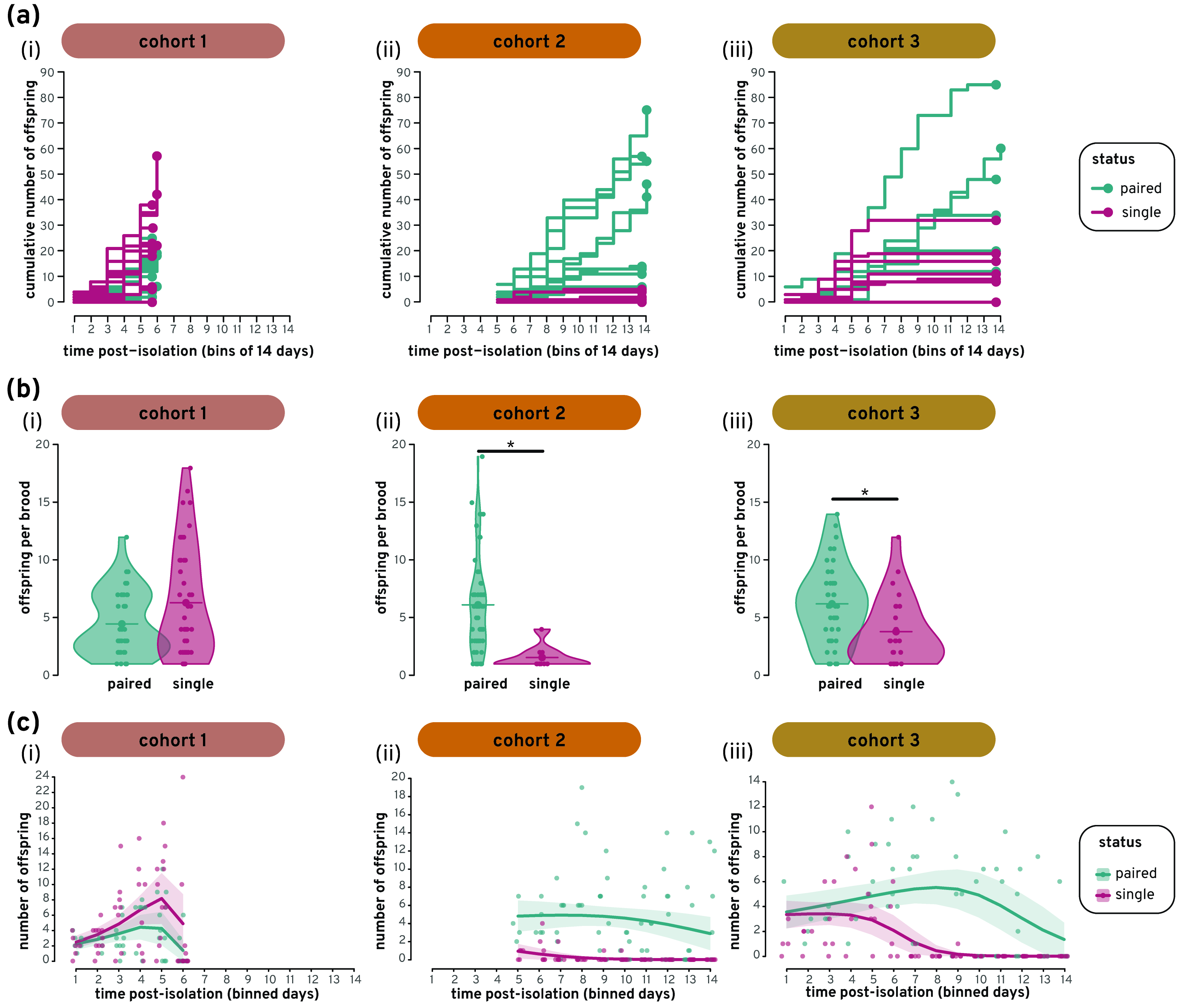

Supplement: Supplementary file 5 — Figure S4: Offspring per brood per cohort: (a) The cumulative number of offspring produced by individual single and paired fish during the birth‐tracking period per cohort: (i) cohort 1, (ii) cohort 2, and (iii) cohort 3. Cumulative sum lines are colored to indicate status (paired or single) and end with a point to indicate the final cumulative sum for each individual fish. (b) The average number of offspring per brood produced by single and paired fish during the birth‐tracking period per cohort: (i) cohort 1, (ii) cohort 2, and (iii) cohort 3. Individual points show the number of offspring per brood data used to generate the violins. The large points with horizontal lines indicate the mean per group. Significance between groups is marked with a star. (c) The number of offspring over time, in binned days post‐isolation, for paired and single fish in each of our cohorts as predicted by our ZINB models, where: (i) cohort 1, (ii) cohort 2, and (iii) cohort 3. Lines indicate the model predictions of the effect of the interaction variable (status (single/paired) × time post‐isolation (binned days)), with the lower and upper bounds of the confidence interval shown by the transparent ribbons. Points on the graphs show the actual number of offspring per fish in each bin used to generate the model. [file ECE3-15-e72086-s002.tif]

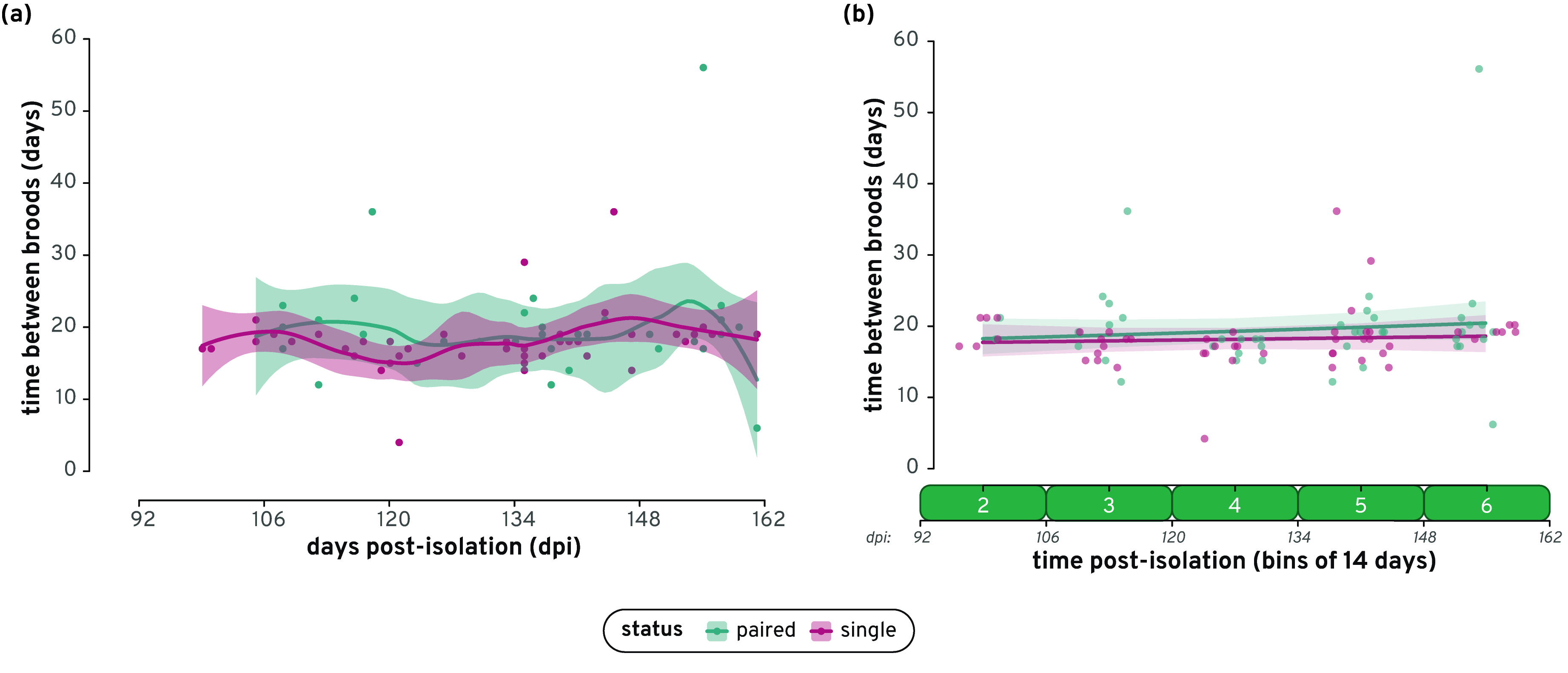

Supplement: Supplementary file 6 — Figure S5: Time between broods (i.e., interbrood interval) over time for bins 1–6: (a) A scatter‐plot of the number of days between broods over time for paired and single fish during bins 1–6. Fitting lines for each group include 95% confidence intervals shown by the transparent ribbons. (b) The time between broods in days post‐isolation, for paired and single fish as predicted by our ZINB model, where only the first 6 bins were analyzed. Lines indicate the model predictions of the effect of the interaction variable (status (single/paired) × time post‐isolation (binned days)), with the lower and upper bounds of the 95% confidence intervals shown by the transparent ribbons. Points on the graph show the actual time in days between broods per fish in each bin used to generate the model. [file ECE3-15-e72086-s004.tif]
