# Supplementary material for: Long‐Term Sperm Storage in a Superfetatious Live‐Bearing Fish (Poeciliopsis gracilis, Poeciliidae)
Source: Ecol Evol. 2025 Sep 4;15(9):e72086. doi: 10.1002/ece3.72086 (PMC12410990; doi:10.1002/ece3.72086)
Supplement: Supplementary file 7 — Table S1: Outcomes from the ZINB models for the number of offspring per brood for each cohort and for all data combined. Time post‐isolation is abbreviated as tpi. Significance is marked with stars where: *p < 0.05, **p < 0.01, and ***p < 0.001. [file ECE3-15-e72086-s008.docx]

**Table S1.** Outcomes from the ZINB models for the number of offspring per brood for each cohort and for all data combined. Time post-isolation is abbreviated as tpi. Significance is marked with stars where: p*<*0.05 is *, p*<*0.01 is ** and p*<*0.001 is ***.

|  | **estimate** | **SE** | **z-value** | **p-value** |  |
| --- | --- | --- | --- | --- | --- |
| 1. **cohort 1**   *Count model coefficients:*  intercept | 0.57 | 0.22 | 2.59 | *<* 0.01 | ** |
| tpi × status (paired) | 0.24 | 0.06 | 3.80 | *<* 0.001 | *** |
| tpi × status (single) | 0.34 | 0.06 | 5.76 | *<* 0.001 | *** |
| log(theta) | 1.14 | 0.30 | 3.81 | *<* 0.001 | *** |
| *Zero-inflation model coefficients:* intercept | -13.52 | 5.34 | -2.53 | *<* 0.05 | * |
| tpi × status (paired) | 2.50 | 0.97 | 2.58 | *<* 0.01 | ** |
| tpi × status (single) | 2.35 | 0.93 | 2.52 | *<* 0.05 | * |
| **(b) cohort 2**  *Count model coefficients:*  intercept | 1.44 | 0.36 | 4.04 | *<* 0.001 | *** |
| tpi × status (paired) | 0.036 | 0.036 | 0.99 | 0.32 |  |
| tpi × status (single) | -0.27 | 0.10 | -2.71 | *<* 0.01 | ** |
| log(theta) | 1.09 | 0.35 | 3.12 | *<* 0.01 | ** |
| *Zero-inflation model coefficients:* intercept | -4.95 | 1.37 | -3.61 | *<* 0.001 | *** |
| tpi × status (paired) | 0.38 | 0.12 | 3.18 | *<* 0.01 | ** |
| tpi × status (single) | 0.66 | 0.20 | 3.36 | *<* 0.001 | *** |
| **(c) cohort 3**  *Count model coefficients:*  intercept | 1.17 | 0.14 | 8.24 | *<* 0.001 | *** |
| tpi × status (paired) | 0.06 | 0.02 | 3.52 | *<* 0.001 | *** |
| tpi × status (single) | 0.10 | 0.04 | 2.52 | *<* 0.05 | * |
| log(theta) | 0.71 | 0.19 | 3.83 | *<* 0.001 | *** |
| *Zero-inflation model coefficients:* intercept | -5.17 | 0.85 | -6.05 | *<* 0.001 | *** |
| tpi × status (paired) | 0.43 | 0.07 | 5.84 | *<* 0.001 | *** |
| tpi × status (single) | 0.86 | 0.13 | 6.53 | *<* 0.001 | *** |
