# Supplementary material for: Long‐Term Sperm Storage in a Superfetatious Live‐Bearing Fish (Poeciliopsis gracilis, Poeciliidae)
Source: Ecol Evol. 2025 Sep 4;15(9):e72086. doi: 10.1002/ece3.72086 (PMC12410990; doi:10.1002/ece3.72086)
Supplement: Supplementary file 8 — Table S2: Outcomes from the GLM model for the time between broods in bins 1–6 for all cohorts combined. Time post‐isolation is abbreviated as tpi. Significance is marked with stars where: *p < 0.05 and ***p < 0.001. [file ECE3-15-e72086-s005.docx]

**Table S2.** Outcomes from the GLM model for the time between broods in bins 1–6 for all cohorts combined. Time post-isolation is abbreviated as tpi. Significance is marked with stars where: p *<* 0.05 is * and p *<* 0.001 is ***.

|  | **estimate** | **SE** | **z-value** | **p-value** |
| --- | --- | --- | --- | --- |
| **Time between broods**  intercept | 0.06 | 0.006 | 9.47 | *<* 0.001 *** |
| tpi × status (paired) | -0.001 | 0.001 | -1.11 | 0.27 |
| tpi × status (single) | -0.0007 | 0.001 | -0.47 | 0.64 |
